# Survival prognosis of newborns from an intensive care unit through the SNAP-PE II risk score

**DOI:** 10.6061/clinics/2020/e1731

**Published:** 2020-08-06

**Authors:** Renato Oliveira Lima, Ana Paula Ribeiro, Yara Juliano, Carolina Nunes França, Patrícia Colombo de Souza

**Affiliations:** I Programa de Pos-Graduacao em Ciencias da Saude, Universidade de Santo Amaro (UNISA), Sao Paulo, SP, BR.; II Faculdade de Medicina FMUSP, Universidade de Sao Paulo, Sao Paulo, SP, BR.

**Keywords:** Hypothermia, Low Weight, Neonatal Intensive Care Units, Newborn, Risk Assessment, SNAP-PE II

## Abstract

**OBJECTIVES:**

Although child mortality has declined significantly in recent decades, the reduction of neonatal mortality remains a major challenge as neonatal mortality represents 2/3 of the mortality rate in this population. The objective of this study was to evaluate the utility of the Score for Neonatal Acute Physiology Perinatal Extension II (SNAP-PE II) score for evaluating the survival prognosis of newborns admitted to a neonatal intensive care unit (NICU).

**METHODS:**

The study design involved an observational cross-sectional retrospective collection, as well as a prospective component. The sample included all newborns admitted to the NICU validated by the SNAP-PE II tool from January 1 to December 31, 2014.

**RESULTS:**

A predominance of young mothers (25.4 years), underwent prenatal care (86.2%), however a considerable percentage (49.4%) of mothers received insufficient medical consultation (less than six consults during their pregnancy). A prevalence of male admissions (62.4%) were noted in the NICU. Premature (61.7%) and underweight (weight <2,500 grams) newborns were also prevalent. The SNAP-PE II score showed an association between the infants who were discharged from the neonatal unit and the non-survivors. An increased prevalence of low birth weight and hypothermia was noted in the group of non-survivors. The mean arterial pressure appears to be a significant risk factor in the newborn group that progressed to death. Hypothermia, mean arterial pressure, and birth weight were the most significant variables associated with death.

**CONCLUSION:**

The SNAP-PE II was a beneficial indicator of neonatal mortality. The prevention of prematurity and hypothermia by improving maternity care and newborn care can decisively influence neonatal mortality.

## INTRODUCTION

Newborn (NB) health is of fundamental importance to the reduction of child mortality, the promotion of a better quality of life, and the reduction of inequality in health (1).

The health of children with very low birth weight (VLBW) is a current focus of interest and concern. NB survival reflects the service structure for pregnant women and newborn infants in various regions and countries. Current studies indicate that neonatal intensive care reduces the rate of neonatal mortality and morbidity, especially in high risk NB groups, including premature babies, patients with severe congenital malformations, and patients with diseases requiring surgery (2).

As an indicator of the living conditions and health of the population, infant mortality in Brazil is progressively declining, although at a slow rate. Specific efforts from the entire society, especially from health workers and prenatal services, are required to accelerate its’ reduction and achieve improved rates for the Brazilian population. Neonatal mortality (between 0 and 27 days of life) comprises approximately 60% to 70% of child mortality, therefore further advances in the health of Brazilian NBs are of significant relevance (3).

The increase in the survival rate of premature infants, especially among infants with VLBW, has been observed in developed, as well as developing countries. The vulnerability of these babies, the risk of dying, and the incidence of sequels resulting from the conditions of their birth highlight the need for monitoring and evaluating their long-term prognoses (2).

Measurements of disease severity and the risk of death in newborns admitted to neonatal intensive care units (NICU) have grown in importance. Risk scores have been used in various situations and allow useful comparisons between countries, times, intensive care units, and treatment groups. A major application of risk scores involves the clarification of unexplained variations in practices and newborn development in dissimilar intensive care units. Risk scores are a useful method to improve the quality of care and assist in planning and monitoring treatment capabilities (4).

After more than a decade, various scores such as the Score for Neonatal Acute Physiology (SNAP) and subsequently, the Score for Neonatal Acute Physiology Perinatal Extension (SNAP-PE), have been proposed for use in assessing birth weight, small-for-gestational age status, and the Apgar score (5). SNAP-II is designed for the measurement of physiologic severity of illness, whereas SNAPPE-II is more appropriate for risk adjustment, as it considers the independent effects of nonphysiologic baseline characteristics. The scores predicted death accurately in a large cohort of 14,610 infants in the original validation (1). Since then, SNAP-II and SNAPPE-II have facilitated the comparison of practices and outcomes in two very large neonatal networks, namely the Canadian Neonatal Network and the Kaiser Permanente network of NICUs in California (2,3). These scores were validated and re-applied in various studies and in various countries (1-5

Given the difficulty in collecting data, the authors produced a simpler version of the SNAP-PE with fewer items to be evaluated. It is termed the SNAP-PE II, and thereby increases the use of the tool, thus increasing the scores assigned to perinatal variables (5,6). Considering this, the objective of this study was to assess the utility of the SNAP-PE II score for evaluating the survival prognosis of newborns admitted to a NICU.

## METHODS

### Design and data collection

We performed a prospective cross-sectional study, that included 209 newborns admitted to the NICU of one Hospital in the city of Itapecerica da Serra in the State of Sao Paulo, Brazil. The following exclusion criteria were applied: congenital malformations incompatible with life; death during the initial 24 hours of admission to the NICU; NICU discharge before completion of 24 hours of admission; patients admitted to the ICU over 12 hours of life and patients with insufficient medical record data that compromised the calculation of scores. The project was approved by the Research Ethics Committee and subjects signed written informed consent for participation in project number: 556051.

### Variables

Mean arterial pressure was measured using Dixtal^®^ and Ranger^®^ oscillometric tensiometers. For several newborns with the need for invasive mechanism pulmonary ventilation associated or not with continuous infusion of vasoactive drugs in the presence of great difficulty in measuring blood pressure using the oscillometric method, mean blood pressure values less than 20 mmHg were considered.

The lowest temperature in degrees Celsius obtained for the study was taken in the axillary region during the first 12 hours of life. The thermometers used were the: Termo MED^®^ and Garatherm Medical AG^®^ brands.

The only examination that was part of the service's routine collection, (that was used for the measurement scores), was arterial blood gases.

The data collected from arterial blood gases were: hydrogen potential serum (pH) and arterial oxygen pressure (PaO2). The relationship between PaO2 and Inspired Oxygen Fraction (FiO2), PaO2 / FiO2 ratio was used to calculate the score.

Patients who developed seizures in the first 12 hours of life were divided into two groups: 1. absence or presence of a single episode, and 2. multiple episodes of seizures.

The diuresis obtained during the first 12 hours of hospitalization was obtained by urine collection with: Cremer^®^ collection bags, Rusch^®^ brand urethral tubes, or the weight of Karícia^®^ brand diapers. For the weight of the diapers, FILIZOLA brand pediatric scales were used. Birth weight in grams was obtained in the delivery room. Patients were weighed by the attending physician responsible for Neonatal Resuscitation or the nurse responsible for that division.

The Gestational Age was obtained through calculation by the Capurro method (21) for patients aged 34 weeks or older, and by the “New Ballard” method (22,23) for patients younger than 34 weeks. A SGA (small for gestational age) newborn was considered to be one whose weight was below the 3% percentile for their gestational age (24).

The Apgar score for the fifth minute of life was established by the physician responsible for the delivery room of each patient.

### Data collection Instrument

The risk of mortality, obtained through the SNAP-PE II, was subsequently calculated by the researcher via the program used by the French Society of Anesthesia and Reanimation. The physiological and laboratory parameters collected within 12 hours of each patient delivered were used to generate a score proportional to the severity of the disease that ranged from 0 to 162 points.

### Statistical analysis

The Mann-Whitney test was used to compare quantitative variables between the death and survivor groups. Fisher's exact test was used to study associations between risk factors and the non-survivor and survivor groups. The significance level was *p*<0.05.

## RESULTS

A predominance of young mothers (25.4 years) underwent prenatal care (86.2%). However, a considerable percentage (49.4%) of mothers received insufficient medical consultation (less than six consults during the pregnancy).

A prevalence of male admissions (62.4%) were noted in the NICU where the study was conducted. Premature (61.7%) and underweight (weight <2,500 grams, median=2217 grams) newborns were also prevalent.

In the assessment of the individual components of the SNAP-PE II score (Table 1), an association was established between the infants who were discharged from the neonatal unit and the non-survivors. The average weight was lower in the non-survivor group (mean=1,654 grams, median=1,100 grams, *p*=0.0028; Mann-Whitney test). In addition, increased hypothermia was observed in the non-survivor group (*p*=0.0093; Mann-Whitney test).

We assessed the SNAP-PE II score components, comparing risk factors among the NB groups (Table 2). An increased prevalence of low birth weight and hypothermia was noted in the group of non-survivors (*p*<0.0001 for both cases, Mann-Whitney test). In Table 2, the mean arterial pressure appears to be a significant risk factor in the newborn group that progressed to death (*p*=0.0002, Mann-Whitney test).

Table 3 presents the concomitance of the variables that form the SNAP-PE II. Hypothermia, mean arterial pressure, and birth weight were the most significant variables associated with death.

## DISCUSSION

The risk scores are based on physiological, subjective and objectives applied to patients admitted to an intensive care unit. The subjective view of health professionals served as the foundation of the scores’ prognoses; a simple observation often served to define the evolution, prognosis and treatment to be adopted (5). With the advancement of medical technology, it was recognized that this subjective view was not enough to direct the treatment of patients. From this deduction, objective criteria began to be more valued, such as clinical data (anamnesis and physical examination), laboratory tests and imaging (15).

The initial versions underwent improvement through mathematical models based on the analysis of a sufficient amount of data gathered from multicenter studies.

The APACHE (Acute Physiology and Chronic Health Evaluation), was created in 1981. It was the first generic prognostic system created for adult patients admitted to intensive care units. Due to the complexity of the original system, a new model was improved in 1985, reducing the number of variables examined and facilitating its’ use. In 1991 there was a further improvement from the study of a sample of 17,440 patients (25).

In the field of Pediatric Intensive Care, some scores have been created and improved over time such as: PSI (Physiologic Stability Index), PRISM (Pediatric Risk of Mortality), PIM (Pediatric Index for Mortality) and a multiple dysfunction assessment model for organs, the PELOD (Pediatric Logistic Organ Dysfunction) (26).

In 1993, there were three neonatal mortality risk scores created for newborns admitted to intensive care units. They were based on models of logistic regression and included clinical and laboratory data from the first 12 to 24 hours of life: CRIB (Clinical Risk Index for Babies), SNAP and SNAP-PE (Score for Neonatal Acute Physiology Perinatal Extension) (27).

In order to increase newborns' survival rates in Neonatal Therapy Units, these prognostic assessment indexes have been studied and improved. The prediction of morbidity and mortality in this age group is based on factors such as weight at birth, gestational age, pregnancy and delivery condition characteristics, children's physiological characteristics and the severity of their illnesses (15).

In Brazil, specific studies on neonatal mortality have become increasingly important considering the increased involvement of the neonatal component in infant deaths. In recent years, the viability of live births has been increasing, and these babies now survive for a longer period. This is through the use of more advanced technology to care for the NB and medicines available for prenatal, perinatal, and postnatal care (7,8). In this study, an increased prevalence of low birth weight and hypothermia were noted in the non-survivor group. Birth weight is an important indicator of the health of the population as it reflects the social, economic, and environmental conditions to which the woman is subject during the gestation period. In addition, low birth weight or being underweight are the main risk factors for the survival of the NB and is predictive of the quality of life.

The mortality of VLBW (birth weight less than 1,500 grams) newborns in 41 Brazilian hospitals that participate in the “Analysis and Intervention for Neonatal Care Improvement” project was 32.7% from 2009 to 2011. This rate increased 2.5-fold compared with the Vermont Oxford Network, which was 12.5% in 2008 with a median of 750 participating institutions (9-12). Data from the Brazilian Network of Neonatal Research indicated that the median survival of these NB was 83.7% in 2010. Data from Child Health and Human Development Neonatal Research indicates a survival rate of 84% in 2008 that increased to 86% in 2009. Various indicators are also better in some South American countries compared with Brazil. There is an urgent need for investment in an appropriate strategic plan that brings together local, state, and federal levels to form a hierarchy and regionalize perinatal care services (12- 13).

Hypothermia in preterm infants is of great concern. This condition occurs frequently and is a risk factor for a poor prognosis, increased morbidity, and neonatal mortality. Thus, strategies to prevent heat loss can impact the morbidity and mortality of NBs, especially preterm NBs, and can improve their prognosis (14). The World Health Organization defines 36.5 to 37°C as a normal range of temperature in NBs and classifies hypothermia according to the severity of the cold exposure: potential cold stress (mild hypothermia, temperature between 36 and 36.4°C); moderate hypothermia (temperature between 32-35.9°C); severe hypothermia (temperature less than 32°C) (14).

Hypothermia leads to decreased surfactant production and increased oxygen consumption. It causes depletion of caloric reserves, contributing to the development or worsening of respiratory failure. In severe hypothermia, hypotension, bradycardia, irregular breathing, decreased activity, weak suck, decreased reflexes, nausea and vomiting, metabolic acidosis, hypoglycemia, hyperkalemia, azotemia, and oliguria may occur. In addition, generalized bleeding, pulmonary hemorrhage, and death have been infrequently noted (14). In our study, the lack of a protocol aimed at thermoregulatory care of the NB, including care in the birth room, during transport, and in the NICU, could have contributed positively to the increased hypothermia observed in the non-survivor group.

Concerning the mean arterial pressure, no consensus is available on the appropriate setting for a premature VLBW infant. The Joint Working Group of the British Association of Perinatal Medicine recommends that the mean arterial pressure in mmHg should be maintained equal to or higher than the gestational age in premature weeks. However, the lack of a protocol for measurements from the catheterization of the umbilical artery as well as the lack of consensus on the normal values for this variable could compromise the results (15). Systemic hypotension is a relatively common complication in preterm NBs with VLBW in the first 72 hours of life and has been associated with mortality, intraventricular hemorrhage, periventricular leukomalacia, and neurodevelopmental morbidity (15,16).

A final important aspect to be addressed is the fact that several studies have indicated that the Apgar score is flawed as the sole criterion for the diagnosis of perinatal asphyxia. Premature NBs have low Apgar scores without presenting fetal acidemia. A significant correlation has been noted between gestational age and Apgar scores in the first and fifth minutes of life. The more premature the NB the greater the probability of a low Apgar score as well as arterial cord blood pH within a normal range (17).

In term NBs, the Apgar score is not trustworthy for the diagnosis of perinatal asphyxia. Thorp and collaborators reported a pH >7.10 in the umbilical arterial blood of 77.8% of NBs with depressed terms (Apgar scores in the 1^st^ or the 5^th^ minute of life <7); (17). The Apgar score is not used to determine the initiation of resuscitation or the interventions to be established during the proceedings. However, its’ application allows assessment of the patient's response to the interventions that are performed and the effectiveness of those interventions. Thus, if the score is below 7 in the 5^th^ minute, it is recommended that these interventions are performed every 5 minutes up to 20 minutes of life. It is necessary to document the Apgar score in a manner that is concomitant with the executed resuscitation procedures (18). The prognosis of premature infants who require advanced resuscitation intervention is more guarded, therefore the presence of health workers who are properly trained to assist this population becomes essential.

Although performed in an environment with the shortest route and an apparent guarantee of safety for the NB, intra-hospital transport may constitute additional risk to the patient. Improving assistance in the birth room to the smallest premature NB (<34 weeks) and adherence to the High Risk Newborn transport protocols could reduce the risk of hypothermia and improve ventilatory support. Thus, this would contribute to the reduction of the SNAP-PE II scores, which subsequently correspond to a reduced risk of death.

According to the study of Sundaram et al. (19), the median SNAP-PE II score was significantly increased in babies who died compared with those who survived. In a study developed by Dammam et al. (20), the authors defined a SNAP-PE II value of greater than 30 as “high” and a risk of death of 28% for the participants. Besides, they showed that a SNAP-PE II score greater than 45 predicted, approximately, a 7-fold more likely death compared with lower SNAP-PE II values. Our group published an editorial in 2019 (28) showing a cut-off point considered “high risk” for mortality based on the SNAP-PE II score. The data were compared with the results presented by Richardson et al. (1), who assessed 755 deaths from a study of 25,280 newborns (2.98%). If we consider a value greater than 30 as “high” as Dammam et al. (20) identified in their study, we observe significant agreement with our study. In 76.9% of those who had a fatal outcome, a score greater than 30 was observed. When we applied the value of 30 as a cut-off in Richardson’s (1) sample, we found that 75.7% of those who died had a score greater than 30, which was similar to the percentage noted in our study. In our study, a high SNAP-PE II score (greater than 30) was noted in 76.9% of those who had a fatal outcome. This finding indicates that the score was a beneficial indicator of mortality in the newborn population.

The limitation of this study was a cross-sectional study design which has its’ own limitations, including response and recall biases, as well as the difficulty of establishing a temporal relationship between the exposures and outcomes.

## CONCLUSION

The SNAP-PE II was a beneficial indicator for neonatal mortality. The prevention of prematurity and hypothermia by improving maternity care and newborn care can decisively influence neonatal mortality.

## AUTHOR CONTRIBUTIONS

Lima RO, Juliano Y and Souza PC were responsible for the manuscript writing, statistical analysis, intellectual concept, and implementation of the entire research project. Ribeiro AP and França CN were responsible for the manuscript writing, review, and intellectual concept.

## Figures and Tables

**Table 1:** Individual values of the components of SNAP-PE II score based on death or survival of newborns.

Variables	Death	Survival	Mann-Whitney test
**Birth weight**	N=13	N=196	
Minimum-Maximum=640-5.120			Z=2.99
Average	1.654	2.365	*p*=0.0028
Median	1.100	2.220	death<survival
**Urine output**	N=13	N=196	
Minimum-Maximum=0-3.0			Z=0.312
Average	1.42	1.49	*p*=0.7546
Median	0.99	1.28	not significant
**pH**	N=12	N=181	
Minimum-Maximum=6.95-7.47			Z=2.134
Averge	7.27	7.32	*p*=0.032
Median	7.30	7.35	death<survival
**Temperature**	N=13	N=196	
Minimum-Maximum=33.0-36.8			Z=2.60
Average	35.2	35.7	*p*=0.0093
Median	35.5	36.0	death<survival
**PaO_2_**/FiO_2_****	N=12	N=181	
Minimum-Maximum=0.42-6.35			Z=0.843
Average	2.61	2.96	*p*=0.399
Median	2.77	2.85	not significant
**Medium arterial pressure**	N=5	N=167	
Minimum-Maximum=<20-93			Z=1.704
Average	36.8	41.9	*p*=0.088
Median	28.0	75.0	not significant
**Apgar5**	N=13	N=195	
Minimum-Maximum=0-10			Z=1.475
Average	7.1	8.2	*p*=0.140
Median	8.0	9.0	not significant

**Table 2:** Risk Factors from SNAP-PE II related to the death or survival of newborns studied.

	Presence				
Risk factors	Yes	No	Total	% Yes	Exact Fisher test
**SGA**					
Death	2	11	13	15.4	*p*=0.9827
Survival	26	170	196	13.2	not significant
**Birth weight**					
Death	6	7	13	46.2	*p*=0.0000
Survival	6	190	196	3.1	death>survival
**Convulsion**					
Death	1	12	13	7.7	*p*=0.9999
Survival	5	191	196	2.5	not significant
**PaO_2_**/FiO_2_****					
Death	5	7	12	41.6	*p*=0.7561
Survival	65	123	188	34.6	not significant
**Temperature**					
Death	10	3	13	76.9	*p*=0.0001
Survival	46	150	196	23.4	death>survival
**MAP**					
Death	10	3	13	76.9	*p*=0.0002
Survival	46	148	194	23.7	death>survival
**pH**					
Death	2	10	12	16.7	*p*=0.1207
Survival	8	173	181	4.4	not significant
**Diuresis**					
Death	5	8	13	38.5	*p*=0.5479
Survival	60	136	196	30.6	not significant
**Apgar5**					
Death	5	8	13	38.5	*p*=0.0076
Survival	18	177	195	9.2	death>survival

SGA: Small for gestational age; MAP: median arterial pressure.

**Table 3:** Concomitance of the components from the risk score SNAP-PE II among newborns that survived (n=163).

Outcomes	N /%
Birth weight	6 / 3.7
Nutritional classification	26 / 15.9
Convulsion	5 / 3.1
Median arterial pressure	46 / 28.2
Temperature	46 / 28.2
pH	8 / 4.9
Diuresis	60 / 36.8
PaO_2_/FiO_2_	65 / 39.8
Apgar 5	18 / 11.1

Cochran G Test; G calculated=178.20; *p*<0.0001*.
